# Supplementary material for: LIFR inhibition enhances the therapeutic efficacy of HDAC inhibitors in triple negative breast cancer
Source: Commun Biol. 2021 Oct 29;4:1235. doi: 10.1038/s42003-021-02741-7 (PMC8556368; doi:10.1038/s42003-021-02741-7)
Supplement: Supplementary file 2 — Supplementary information [file 42003_2021_2741_MOESM2_ESM.pdf]

Supplementary Figure 1

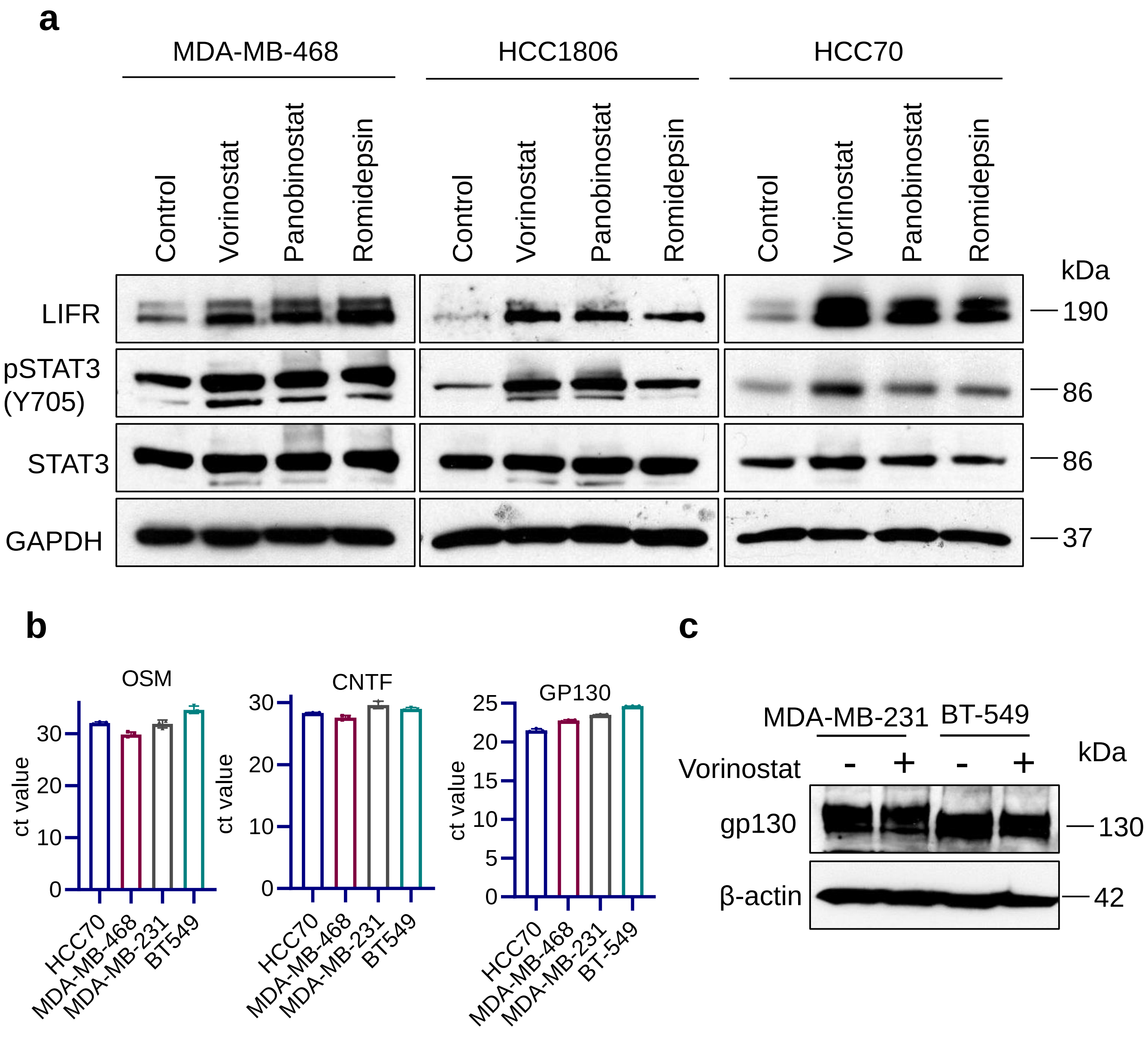

Supplementary Figure 1. HDACi treatment induced STAT3 activation via LIFR expression. **a**. TNBC model cells (MDA-MB-468, HCC1806 and HCC70) were treated with indicated HDACi (vorinostat:10 µM; panobinostat:1 µM; romidepsin:1 µM) for 24 h and expression of LIFR, p-STAT3(Y705) and STAT3 were determined using Western blotting. **b**. Expression of LIFR ligands (OSM and CNTF) and GP130 in 4 different TNBC model cells were analyzed by RT-qPCR. **c**. MDA-MB-231 and BT-549 cells were treated with HDACi and the expression of gp130 was determined using Western blotting.

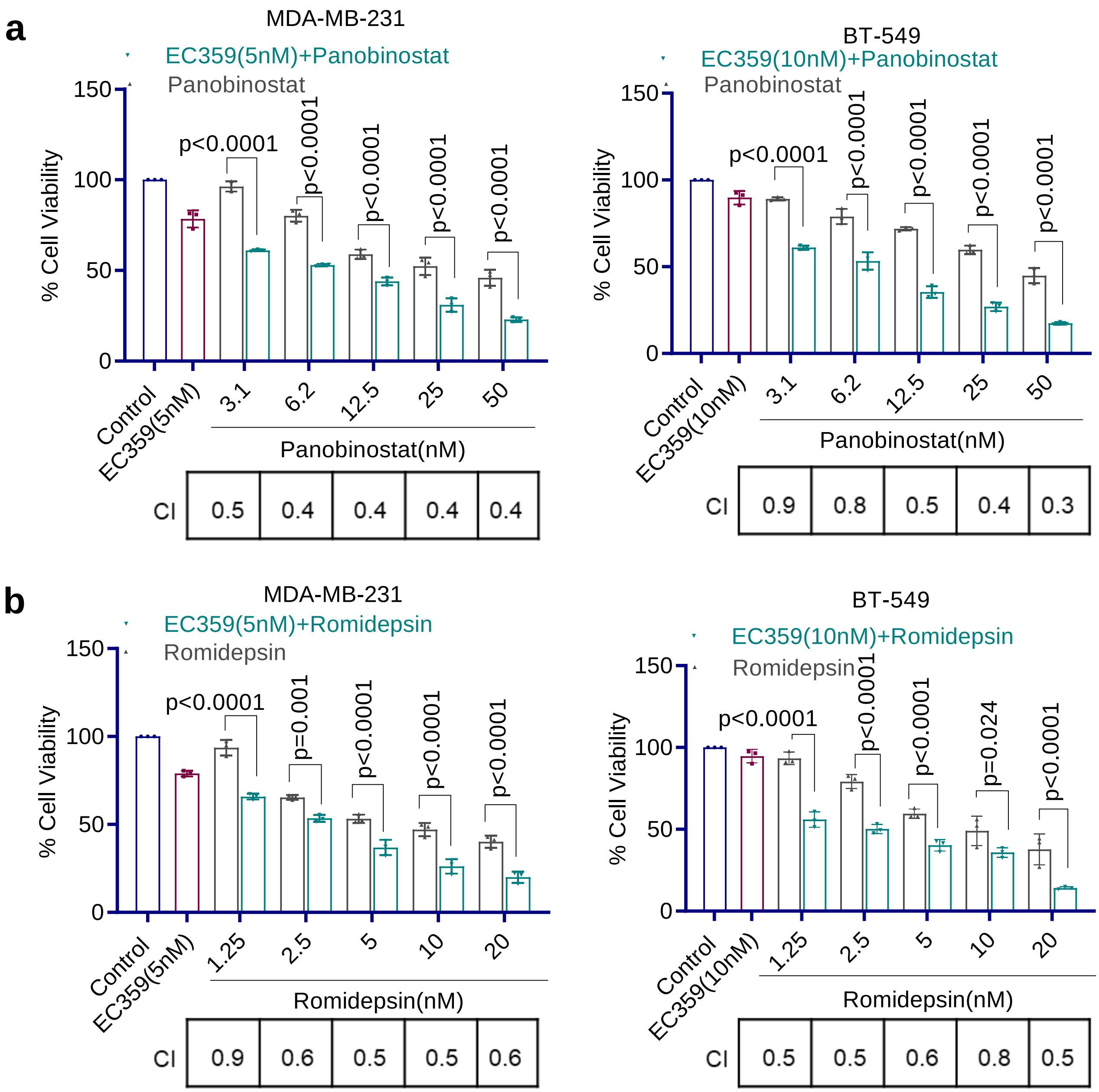

Supplementary Figure 2. EC359 synergistically enhanced HDACi ability to reduce cell viability. MDA-MB-231 and BT-549 cells were treated with indicated concentrations of panobinostat (**a**) and romidepsin (**b**) for 72 h in the presence or absence of EC359 (MDA-MB-231:5 nM; BT-549:10 nM) and the cell viability was measured by MTT assay. Data are representative of 3 independent experiments (n=3). Error bars represent SD. In **a**, and **b**, p-values were calculated using Two-way ANOVA. The combination index (CI) of EC359+HDACi therapy was determined using Chou-Talalay method.

Supplementary Figure 3

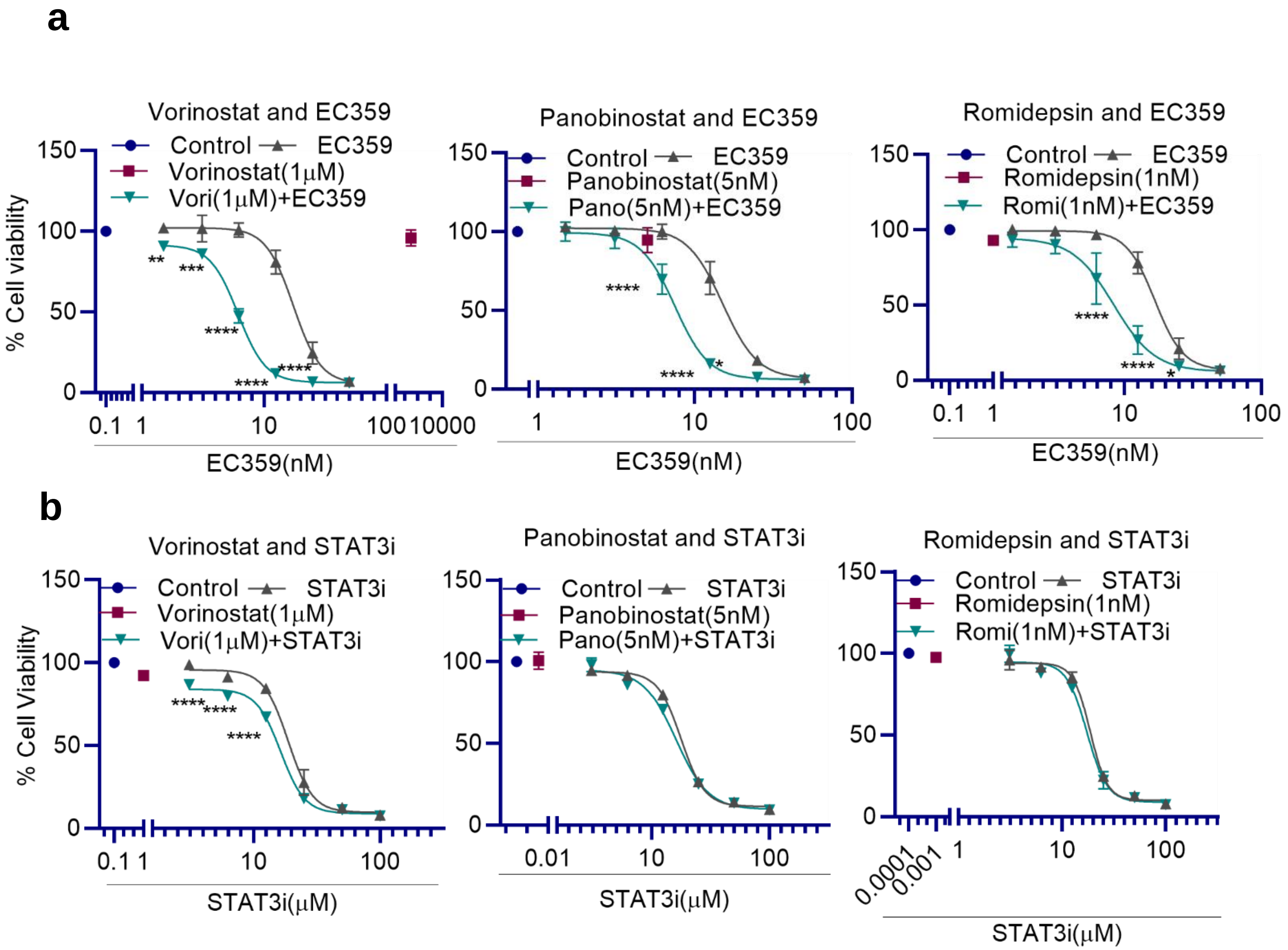

Supplementary Figure 3. EC359 is more effective in potentiating HDACi mediated reduction of cell viability. BT-549 cells were treated with HDACi in combination with EC359 (a) or STAT3i (b) for 72 h and the cell viability was measured using MTT assay. Data are representative of 3 independent experiments (n=3). Error bars represent SD. In **a** and **b**, p-values were calculated using Two-way ANOVA. * p<0.0, ** p<0.01, *** p<0.001, ****p<0.0001.

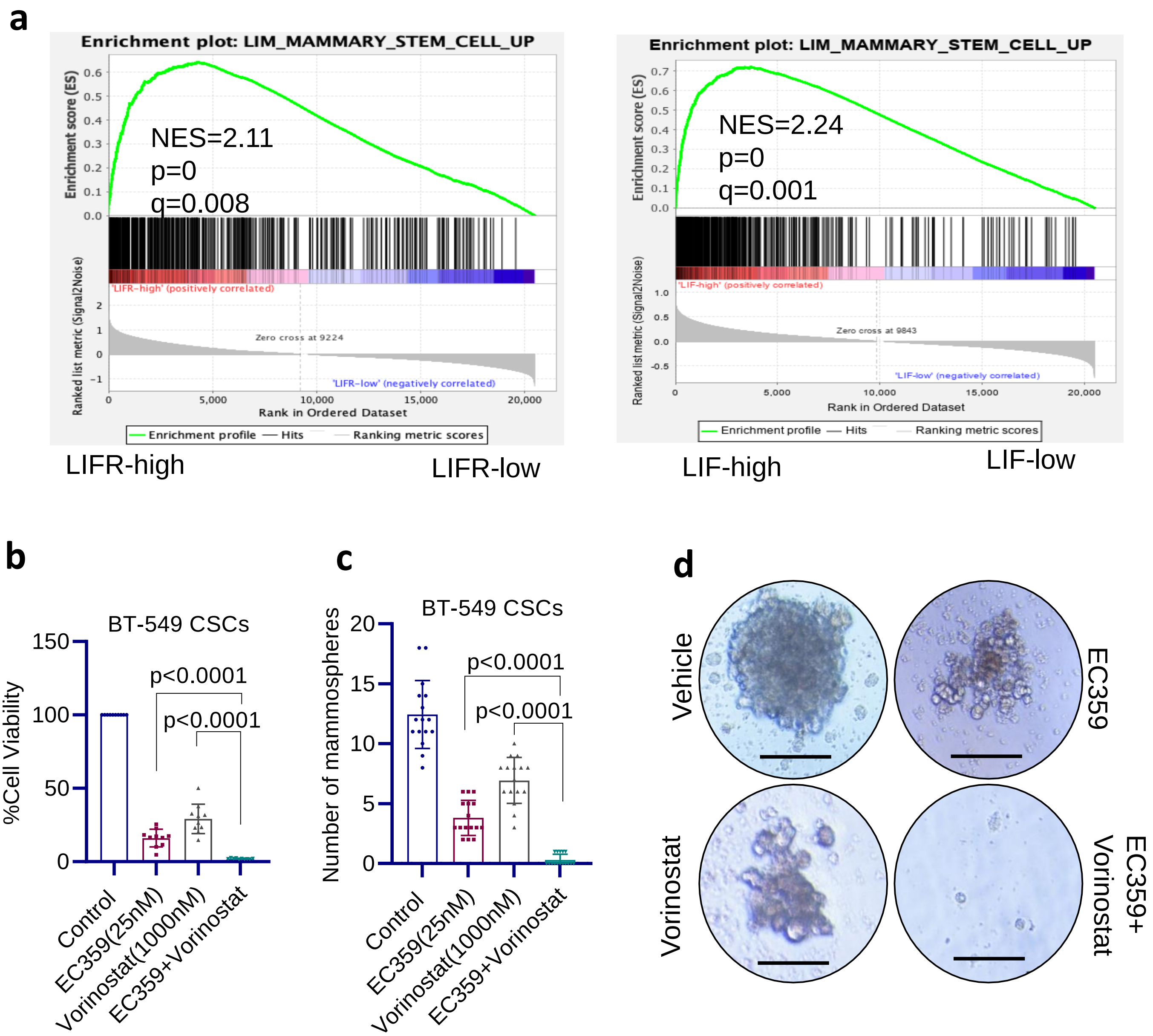

Supplementary Figure 4 a. High expression of LIFR and LIF correlates with the expression of mammary stem cell gene set in TCGA data base. Effect of EC359+vorinostat on the proliferation of CSCs was studied using CellTiter-Glo (n=10) (**b**), and sphere forming ability was studied using mammosphere formation assays (n=16) (**c**). Representative images of mammospheres after treatment were shown (**d**). Scale bar represent 100µm. Error bars represent SD.

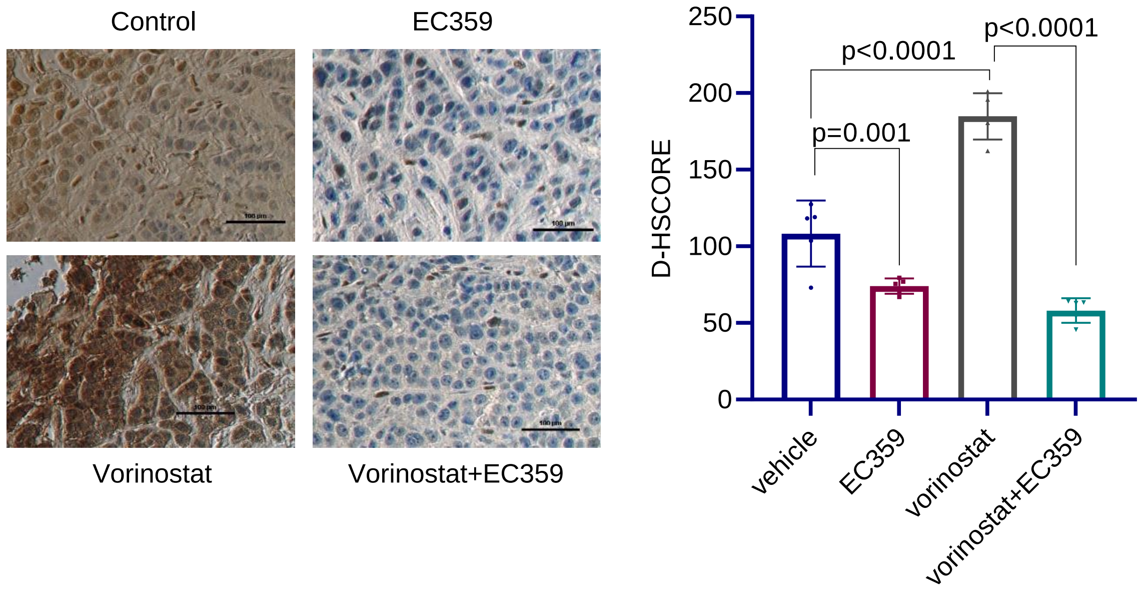

Supplementary Figure 5. MDA-MB-468 xenograft tumors collected from vehicle or EC359 or Vorinostat or combination treated mice were subjected to IHC staining of p-STAT3(Y705). Quantitation of IHC images were done by Image J software. Error bars represent SD. p-value was calculated using one-way ANOVA.

Supplementary Figure 6

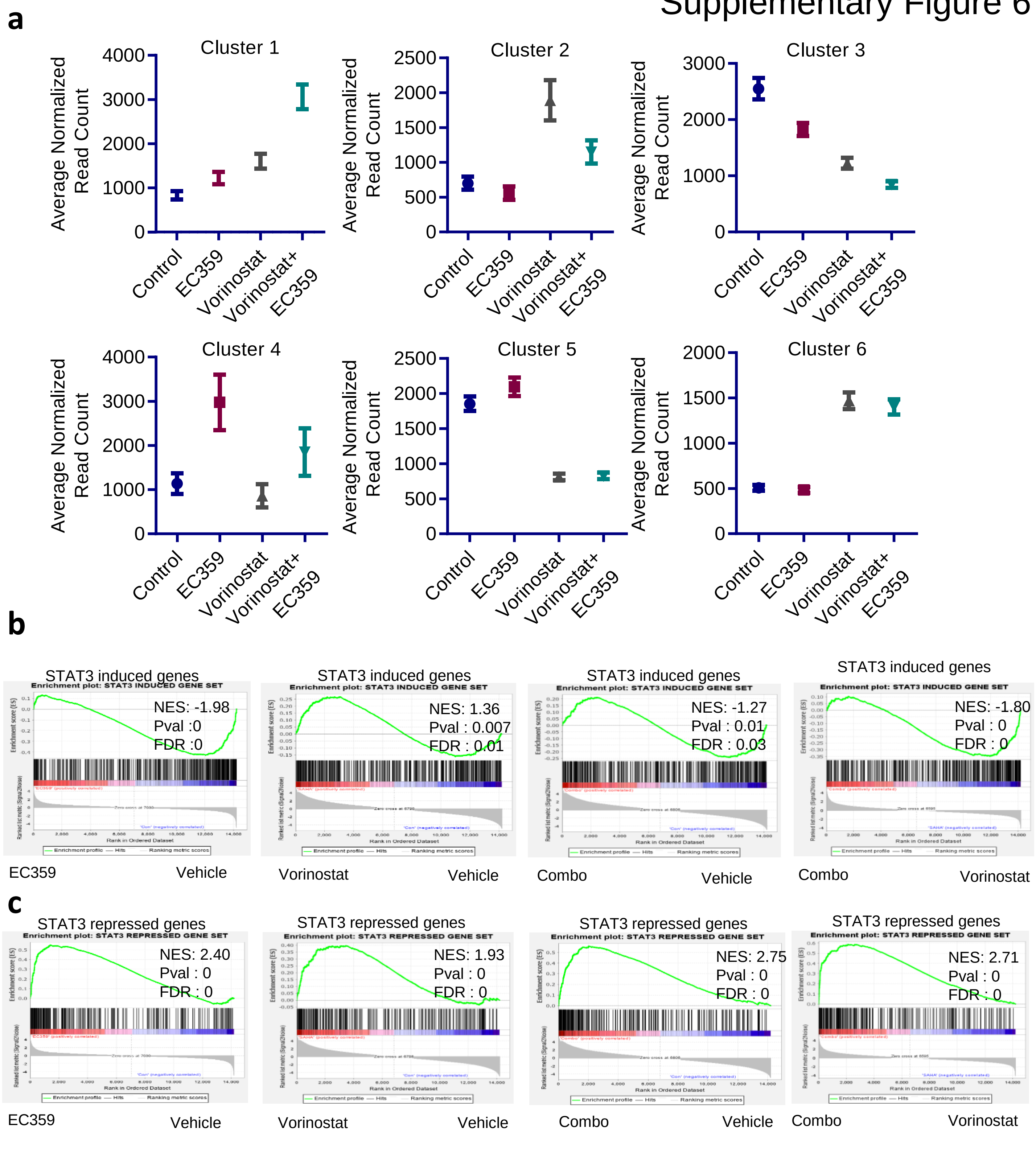

Supplementary Figure 6. a, The differentially expressed genes were grouped into clusters based on their expression pattern across 4 conditions (Control, EC359, Vorinostat, Vorinostat+EC359 (combo)) and six distinct clusters identified (clusters 1–6) are shown. **b, c**, GSEA of STAT3 induced and repressed gene signature between vehicle and EC359, vehicle and Vorinostat, vehicle and combination group and Vorinostat and combo was shown.

Supplementary Figure 7

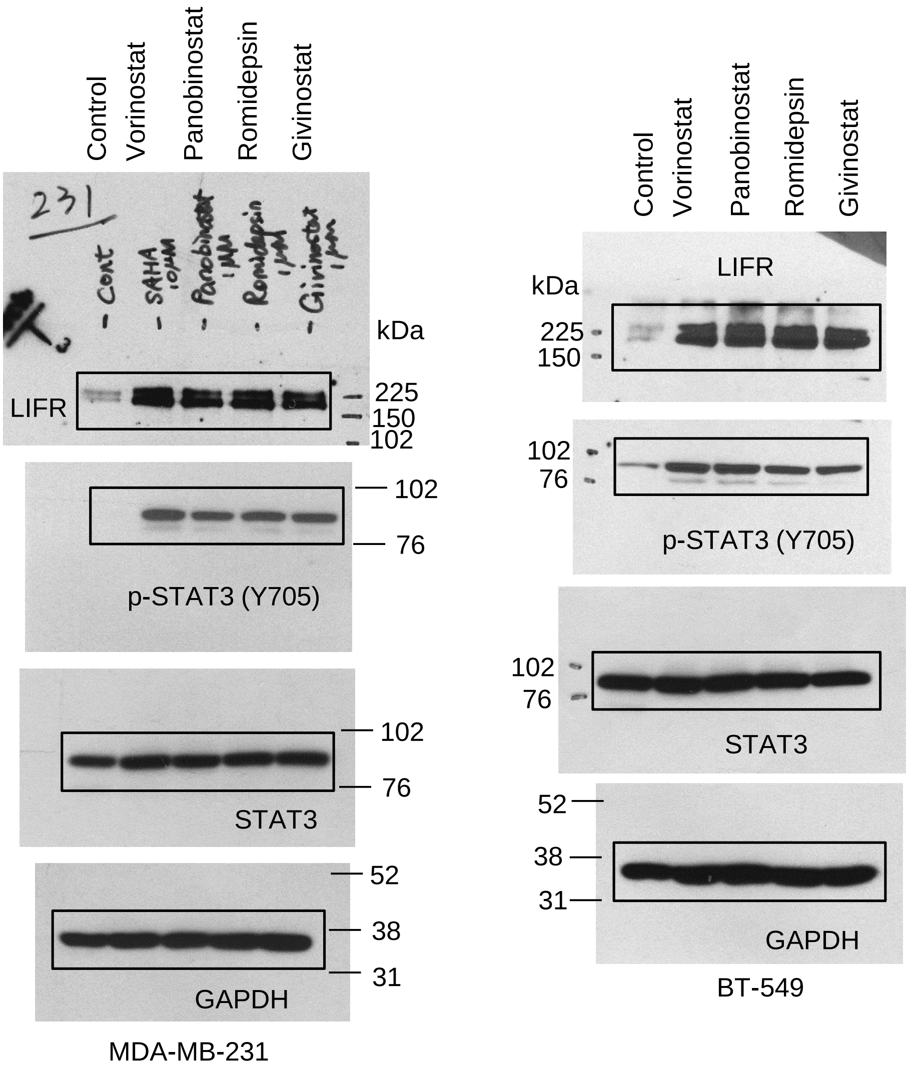

Supplementary Figure 7. Unprocessed scans of Western blots used in Figure 1 a. Cropped sections used as figures in the manuscript are marked as a box.

Supplementary Figure 8

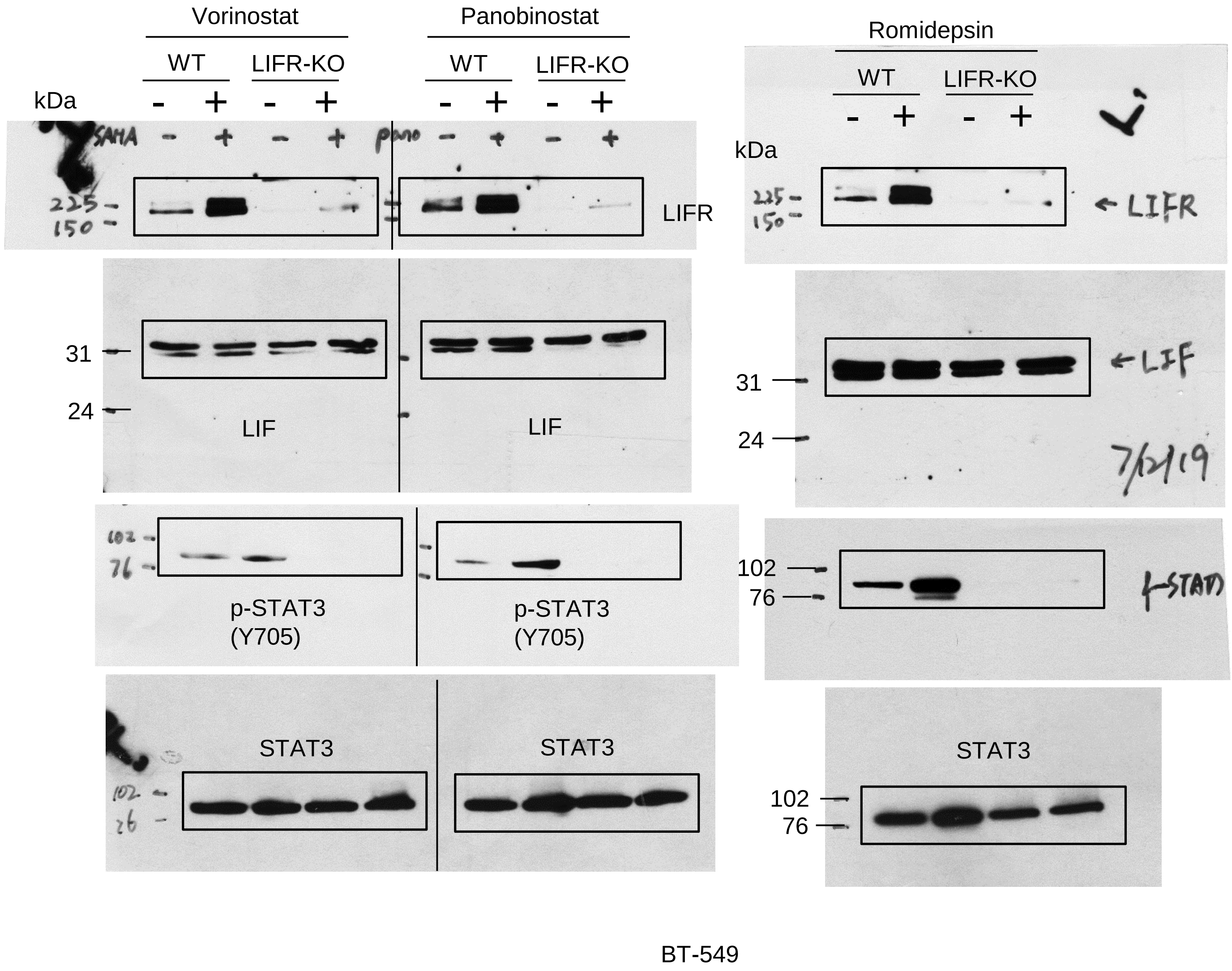

Supplementary Figure 8. Unprocessed scans of Western blots used in Figure 1 d. Cropped sections used as figures in the manuscript are marked as a box.

Supplementary Figure 9

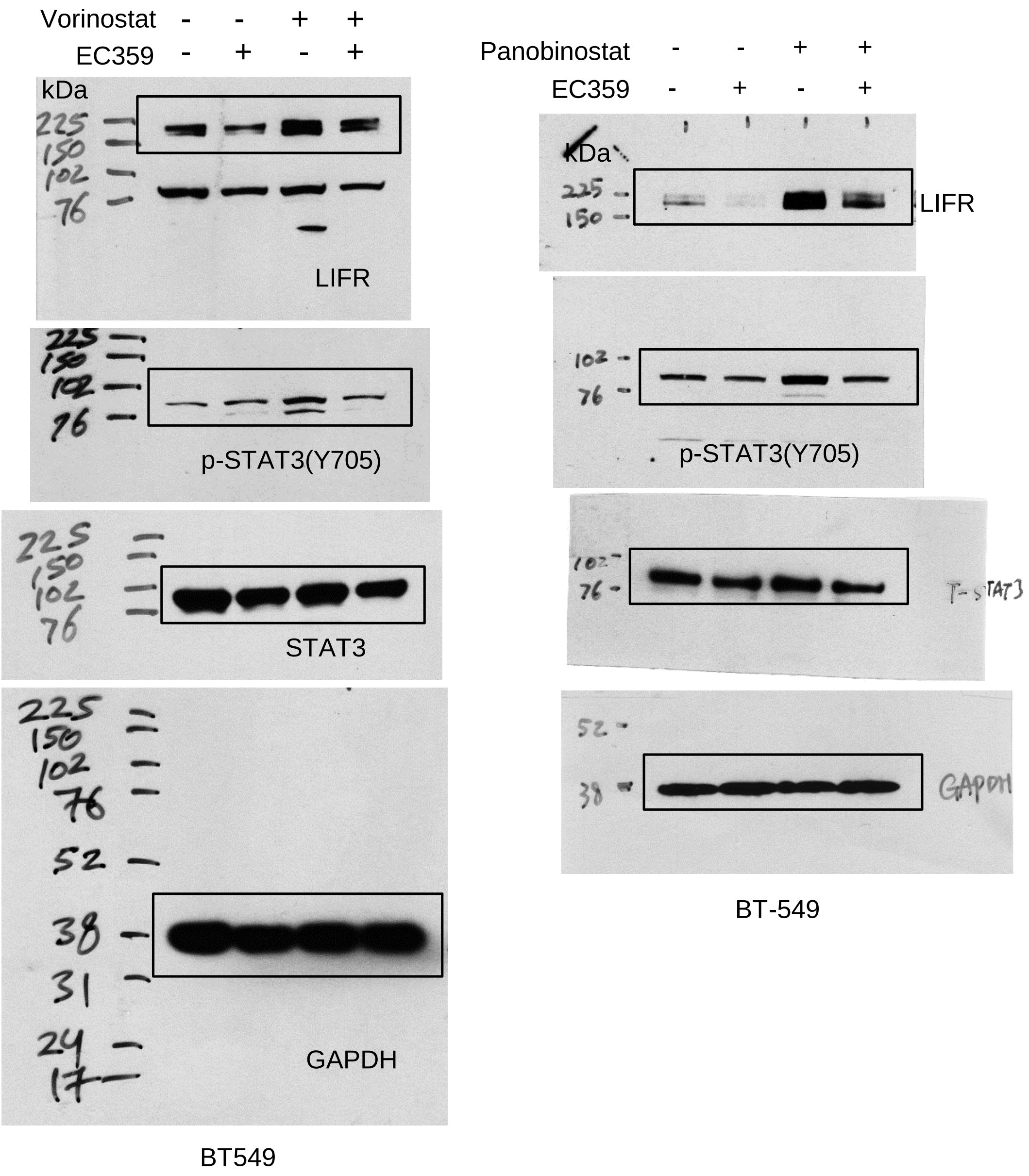

Supplementary Figure 9. Unprocessed scans of Western blots used in Figure 4a. Cropped sections used as figures in the manuscript are marked as a box.

Supplementary Figure 10

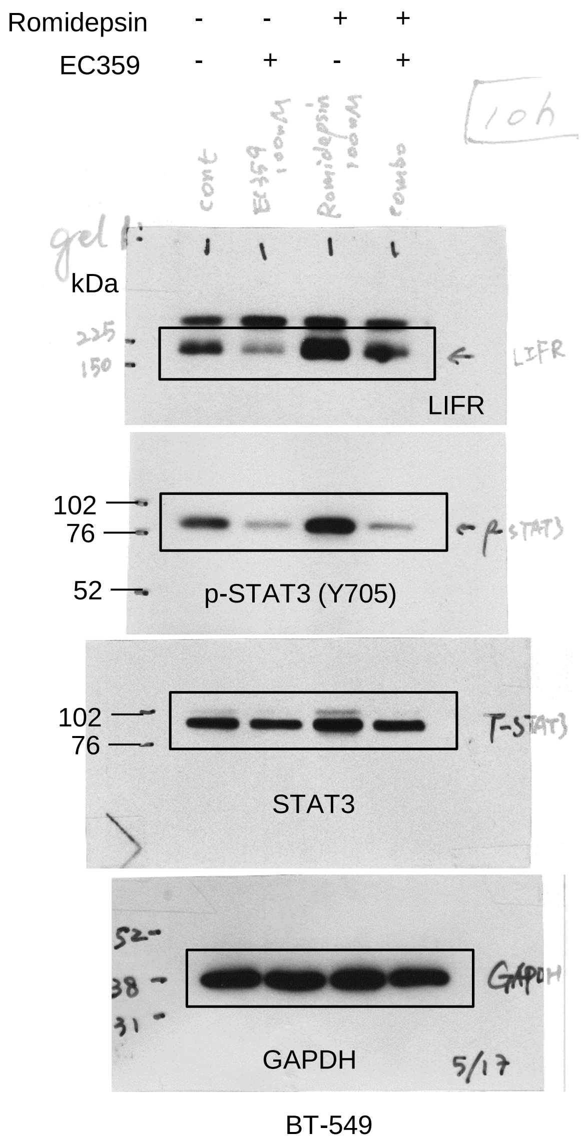

Supplementary Figure 10. Unprocessed scans of Western blots used in Figure 4a. Cropped sections used as figures in the manuscript are marked as a box.

Supplementary Figure 11

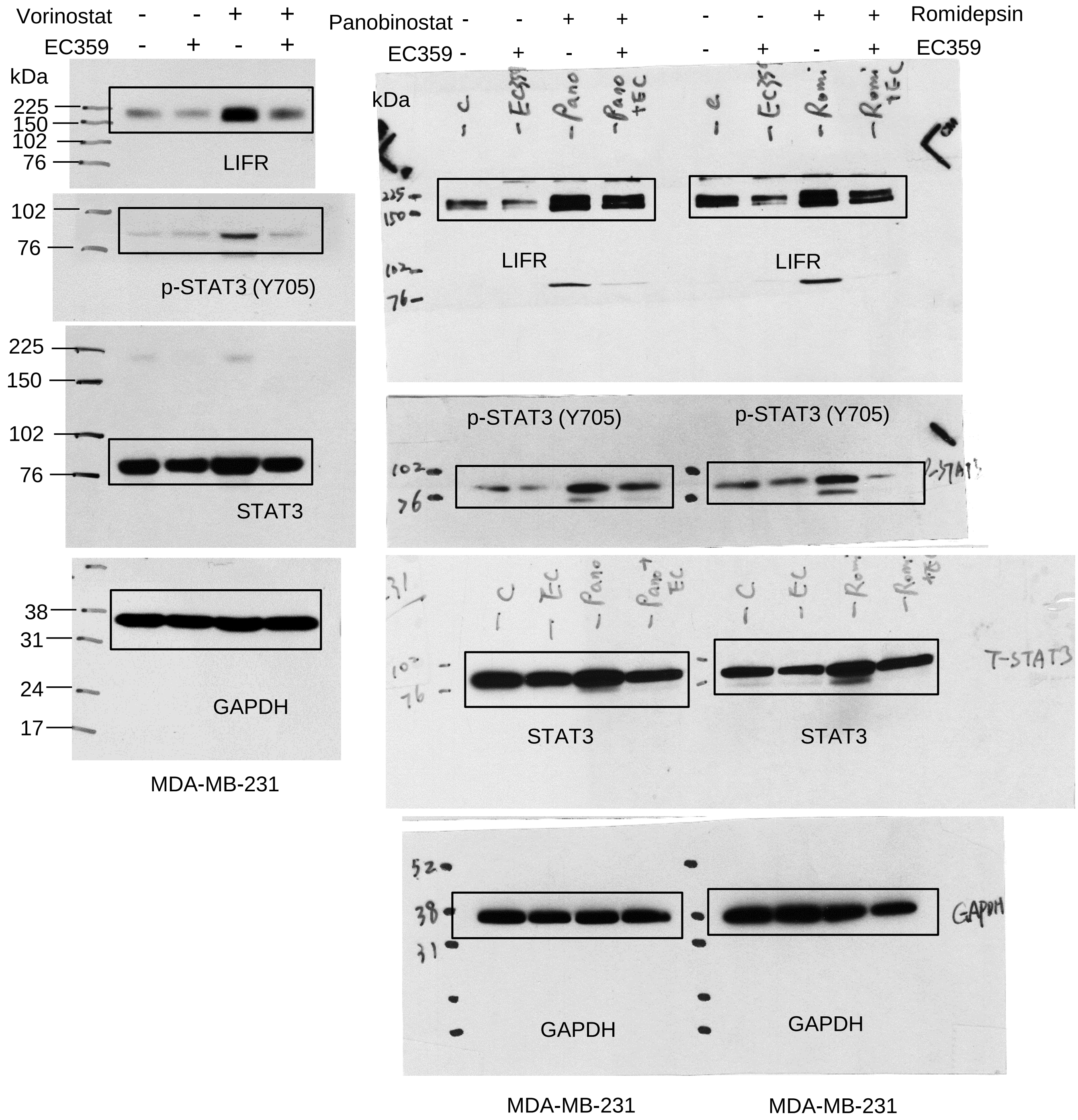

Supplementary Figure 11. Unprocessed scans of Western blots used in Figure 4a. Cropped sections used as figures in the manuscript are marked as a box.

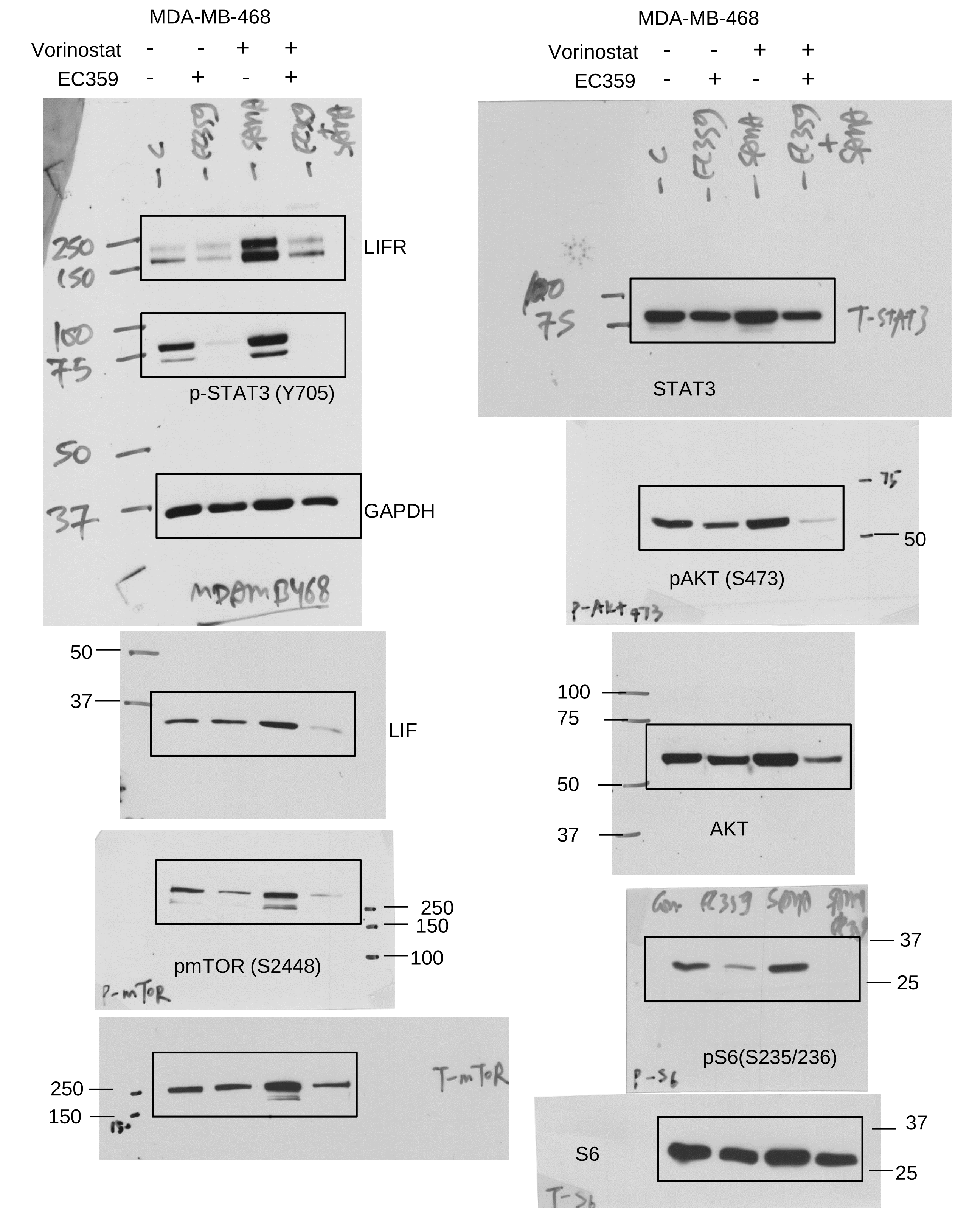

Supplementary Figure 12. Unprocessed scans of Western blots used in Figure 4b. Cropped sections used as figures in the manuscript are marked as a box.

Supplementary Figure 13

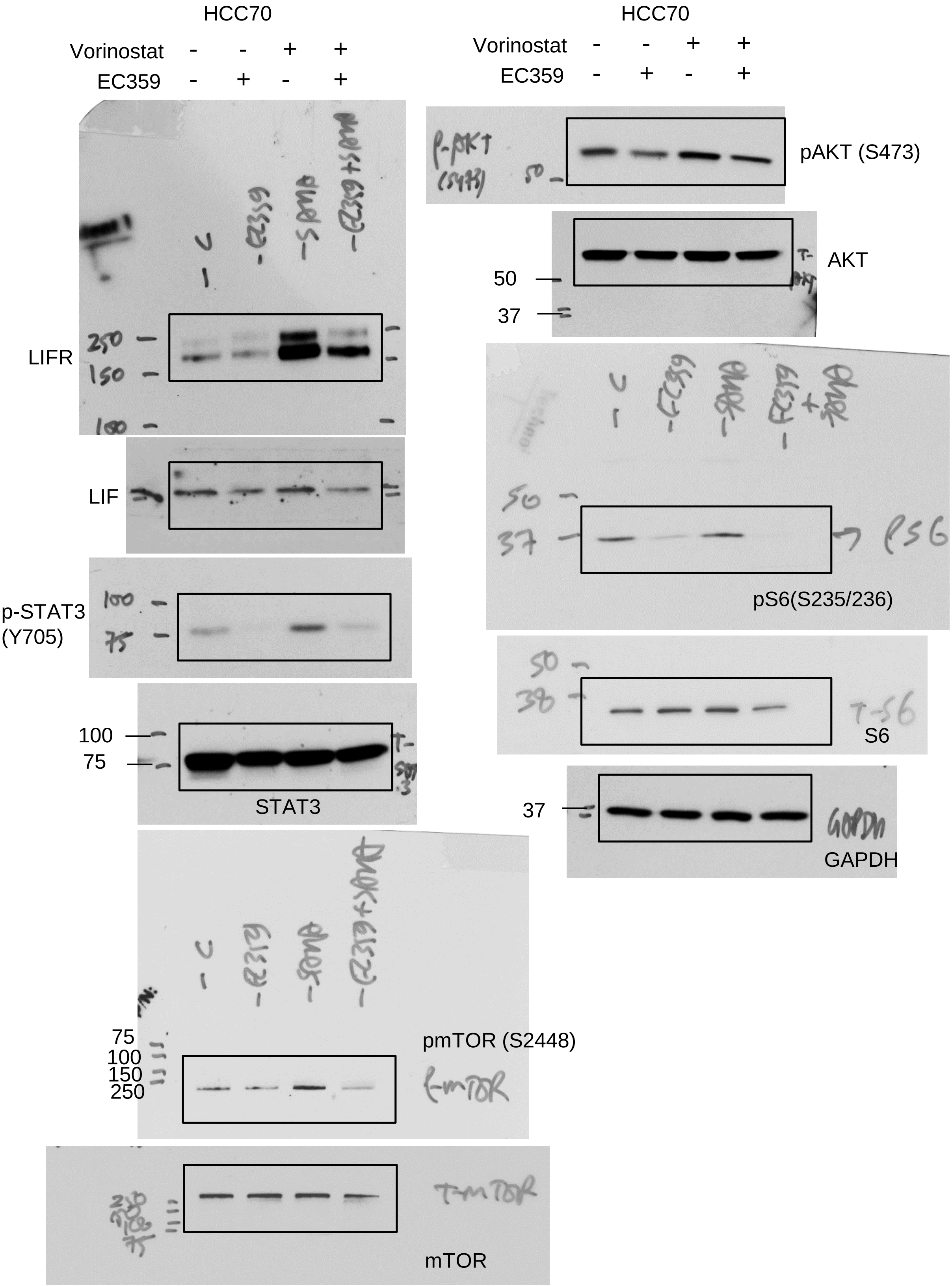

Supplementary Figure 13. Unprocessed scans of Western blots used in Figure 4b. Cropped sections used as figures in the manuscript are marked as a box.

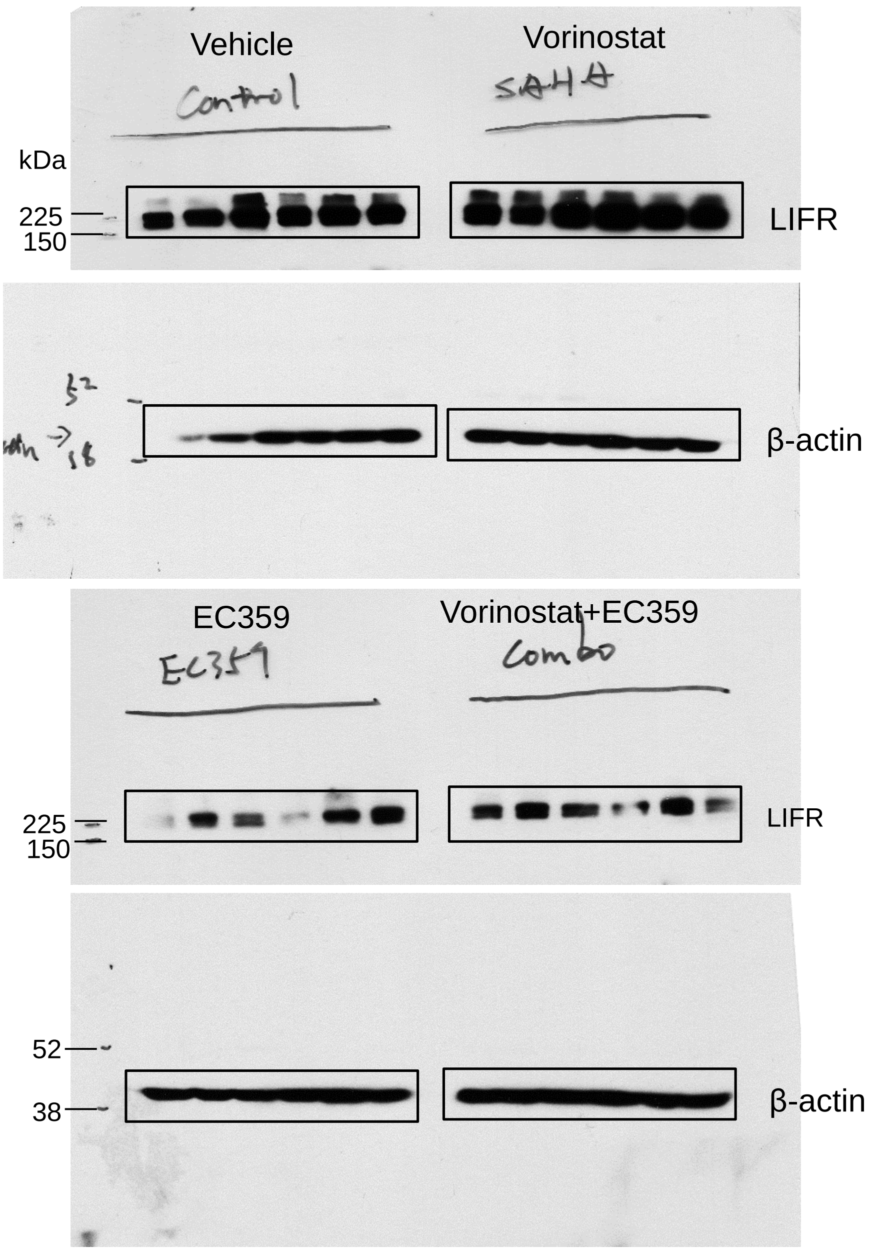

Supplementary Figure 14. Unprocessed scans of Western blots used in Figure 7c. Cropped sections used as figures in the manuscript are marked as a box.

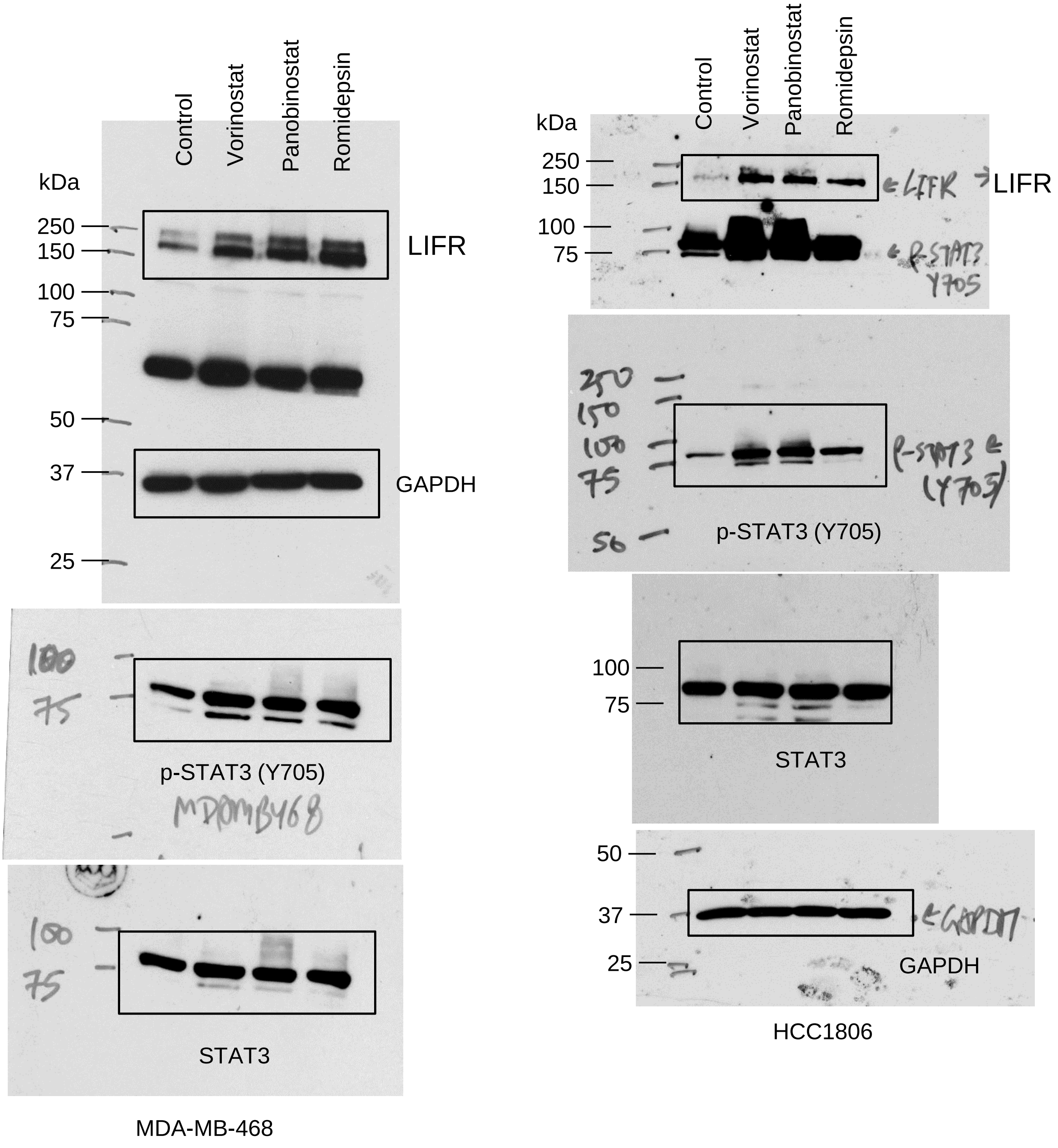

Supplementary Figure 15. Unprocessed scans of Western blots used in Supplementary Figure 1a. Cropped sections used as figures in the manuscript are marked as a box.

Supplementary Figure 16

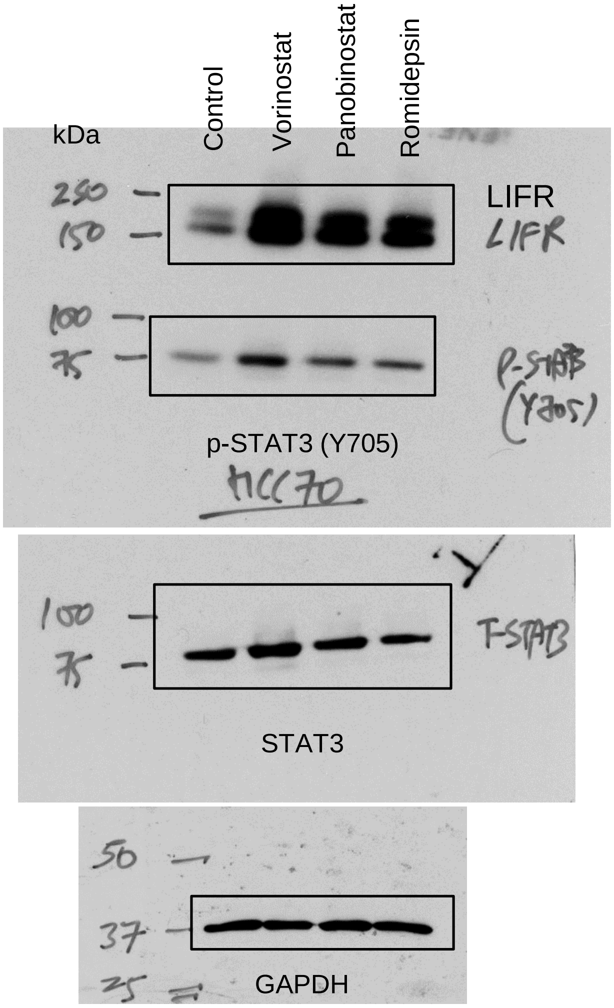

HCC70

Supplementary Figure 16. Unprocessed scans of Western blots used in Supplementary Figure 1a. Cropped sections used as figures in the manuscript are marked as a box.

Supplementary Figure 17

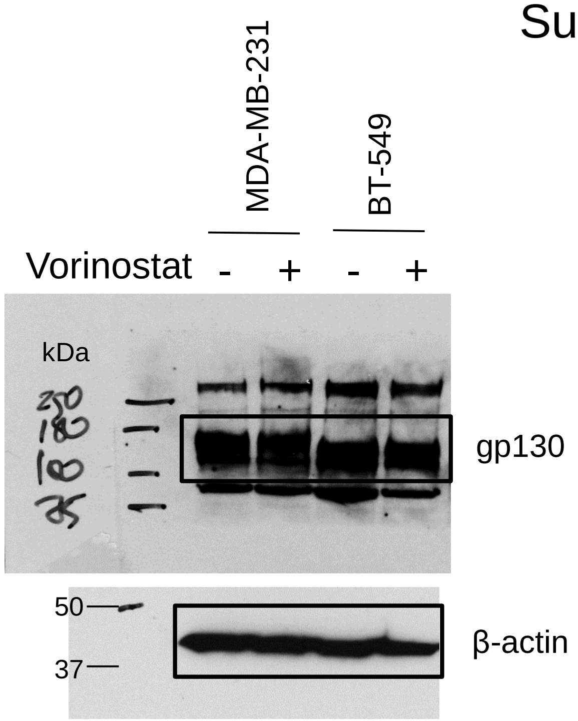

Supplementary Figure 17. Unprocessed scans of Western blots used in supplementary figure 1c. Cropped sections used as figures in the manuscript are marked as a box.

Supplementary table 1

Primer sequences used for RT-qPCR analysis

Gene name	Forward Primer	Reverse Primer
LIFR	TGTCAGGCGTTCTCGTCTC	GAGTTGTGTTGTGGGTCACTAA
CDKN1A	TGTCCGTCAGAACCCATGC	AAAGTCGAAGTTCCATCGCTC
ATF3	CCTCTGCGCTGGAATCAGTC	TTCTTTCTCGTCGCCTCTTTTT
GADD45A	GAGAGCAGAAGACCGAAAGGA	CACAACACCACGTTATCGGG
TNFRSF10B	ATGGAACAACGGGGACAGAAC	CTGCTGGGGAGCTAGGTCT
ID1	CTGCTCTACGACATGAACGG	GAAGGTCCCTGATGTAGTCGAT
ID2	AGTCCCGTGAGGTCCGTTAG	AGTCGTTCATGTTGTATAGCAGG
ID3	GAGAGGCACTCAGCTTAGCC	TCCTTTTGTCGTTGGAGATGAC
UBE2S	ACAAGGAGGTGACGACACTGA	CCACGTTCGGGTGGAAGAT
AURKA	GAGGTCCAAAACGTGTTCTCG	ACAGGATGAGGTACACTGGTTG
CCNB	AATAAGGCGAAGATCAACATGGC	TTTGTTACCAATGTCCCCAAGAG
KIF2C	CTGTTTCCCGGTCTCGCTATC	AGAAGCTGTAAGAGTTCTGGGT
GP130	CGGACAGCTTGAACAGAATGT	ACCATCCCACTCACACCTCA
OSM	CACAGACTGGCCGACTTAGAG	AGTCCTCGATGTTCAGCCCA
CNTF	GAAGATTCGTTCAGACCTGACTG	AAGGTTCTCTTGGAGTCGCTC
CDK4	ATGGCTACCTCTCGATATGAGC	CATTGGGGACTCTCACACTCT
GAPDH	TCGACAGTCAGCCGCATCT	CTAGCCTCCCGGGTTTCTCT
β-actin	GTGGGCATGGGTCAGAAG	TCCATCACGATGCCAGTG
