# Supplementary material for: LIFR inhibition enhances the therapeutic efficacy of HDAC inhibitors in triple negative breast cancer
Source: Commun Biol. 2021 Oct 29;4:1235. doi: 10.1038/s42003-021-02741-7 (PMC8556368; doi:10.1038/s42003-021-02741-7)
Supplement: Supplementary file 3 — Description of Additional Supplementary Files [file 42003_2021_2741_MOESM3_ESM.pdf]

Description of Additional Supplementary Files

File name: Supplementary Data 1.
Description: Source data for the graphs and charts.
