# Supplementary material for: LIFR inhibition enhances the therapeutic efficacy of HDAC inhibitors in triple negative breast cancer
Source: Commun Biol. 2021 Oct 29;4:1235. doi: 10.1038/s42003-021-02741-7 (PMC8556368; doi:10.1038/s42003-021-02741-7)
Supplement: Supplementary file 5 — Reporting Summary [file 42003_2021_2741_MOESM5_ESM.pdf]

nature research

Corresponding author(s): Ratna k Vadlamudi

Last updated by author(s): 09/23/2021

Reporting Summary

Nature Research wishes to improve the reproducibility of the work that we publish. This form provides structure for consistency and transparency in reporting. For further information on Nature Research policies, see our Editorial Policies and the Editorial Policy Checklist.

Statistics

For all statistical analyses, confirm that the following items are present in the figure legend, table legend, main text, or Methods section.

n/a	Confirmed	
☐	☒	The exact sample size (*n*) for each experimental group/condition, given as a discrete number and unit of measurement
☐	☒	A statement on whether measurements were taken from distinct samples or whether the same sample was measured repeatedly
☐	☒	The statistical test(s) used AND whether they are one- or two-sided<br>*Only common tests should be described solely by name; describe more complex techniques in the Methods section.*
☒	☐	A description of all covariates tested
☐	☒	A description of any assumptions or corrections, such as tests of normality and adjustment for multiple comparisons
☐	☒	A full description of the statistical parameters including central tendency (e.g. means) or other basic estimates (e.g. regression coefficient) AND variation (e.g. standard deviation) or associated estimates of uncertainty (e.g. confidence intervals)
☐	☒	For null hypothesis testing, the test statistic (e.g. *F*, *t*, *r*) with confidence intervals, effect sizes, degrees of freedom and *P* value noted<br>*Give P values as exact values whenever suitable.*
☒	☐	For Bayesian analysis, information on the choice of priors and Markov chain Monte Carlo settings
☒	☐	For hierarchical and complex designs, identification of the appropriate level for tests and full reporting of outcomes
☐	☒	Estimates of effect sizes (e.g. Cohen's *d*, Pearson's *r*), indicating how they were calculated

Our web collection on statistics for biologists contains articles on many of the points above.

Software and code

Policy information about availability of computer code

Data collection	The raw reads were aligned to the reference human genome (UCSC hg19) with TopHat2(19) . Genes were annotated (using NCBI RefSeq) and quantified by HTSeq(20) , and DESeq(21) was used to identify differentially expressed genes and significant genes with fold change > 1 and multiple-test adjusted p value <0.01 were used for interpreting the biological pathways.
Data analysis	Gene Set Enrichment Analysis was performed using stand-alone distribution (http://www.gsea-msigdb.org/gsea/index.jsp)

For manuscripts utilizing custom algorithms or software that are central to the research but not yet described in published literature, software must be made available to editors and reviewers. We strongly encourage code deposition in a community repository (e.g. GitHub). See the Nature Research guidelines for submitting code & software for further information.

Data

Policy information about availability of data

All manuscripts must include a data availability statement. This statement should provide the following information, where applicable:

- Accession codes, unique identifiers, or web links for publicly available datasets
- A list of figures that have associated raw data
- A description of any restrictions on data availability

The data supporting the findings of this study are available from the corresponding authors upon reasonable request. Source data for the graphs and charts are provided in the Supplementary Data 1. The RNAseq data has been deposited in the GEO database (accession number GSE163249). Unedited western blot images are available in the Supplementary Information (Supplementary Figures 7–17).

Field-specific reporting

Please select the one below that is the best fit for your research. If you are not sure, read the appropriate sections before making your selection.

☒ Life sciences ☐ Behavioural & social sciences ☐ Ecological, evolutionary & environmental sciences

For a reference copy of the document with all sections, see nature.com/documents/nr-reporting-summary-flat.pdf

Life sciences study design

All studies must disclose on these points even when the disclosure is negative.

Sample size	For animal studies, sample size of tumors/treatment was derived using effect information from previous studies and calculations were based on a model of unpaired data power =0.8; p<0.05.
Data exclusions	No data was excluded
Replication	All in vitro assays were performed in triplicate and repeated at least three times
Randomization	Animal groups were randomized based on tumor volume. All in vitro assays were done in triplicate with no randomization.
Blinding	All in vitro assays were performed in triplicate and repeated at least three times. Investigator was not blinded. IHC studies are blinded for quantitation by independent evalutions.

Reporting for specific materials, systems and methods

We require information from authors about some types of materials, experimental systems and methods used in many studies. Here, indicate whether each material, system or method listed is relevant to your study. If you are not sure if a list item applies to your research, read the appropriate section before selecting a response.

Materials & experimental systems

n/a	Involved in the study	
☐	☒	Antibodies
☐	☒	Eukaryotic cell lines
☒	☐	Palaeontology and archaeology
☐	☒	Animals and other organisms
☒	☐	Human research participants
☒	☐	Clinical data
☒	☐	Dual use research of concern

Methods

n/a	Involved in the study	
☒	☐	ChIP-seq
☒	☐	Flow cytometry
☒	☐	MRI-based neuroimaging

Antibodies

Antibodies used	Antibodies for GAPDH, p-S6, S6, p-Akt (S473), Akt, p-mTOR (S2448), mTOR, p-STAT3 (Y705), and STAT3 were purchased from Cell Signaling Technology (Danvers MA). LIF and LIFR antibodies were purchased from Santa Cruz Biotechnology (Dallas, TX). β-actin antibody was obtained from Sigma-Aldrich (St. Louis, MO). The Ki67 antibody was purchased from Abcam (Cambridge, MA).
Validation	All the antibodies used were commercially validated antibodies and from respectable companies.

Eukaryotic cell lines

Policy information about cell lines

Cell line source(s)	ATCC
Authentication	STR DNA profiling was used to confirm cell identity.
Mycoplasma contamination	All the model cells utilized were free of mycoplasma contamination
Commonly misidentified lines (See ICLAC register)	None used

Animals and other organisms

Policy information about studies involving animals; ARRIVE guidelines recommended for reporting animal research

Laboratory animals	female, athymic nude mice , SCID mice
Wild animals	None
Field-collected samples	None
Ethics oversight	All animal experiments were performed using UTHSA IACUC approved protocol

Note that full information on the approval of the study protocol must also be provided in the manuscript.
